# Research progress in mechanism of anticancer action of shikonin targeting reactive oxygen species

**DOI:** 10.3389/fphar.2024.1416781

**Published:** 2024-07-11

**Authors:** Ke Qi, Jiayi Li, Yang Hu, Yiyun Qiao, Yongping Mu

**Affiliations:** ^1^ Department of Diagnostic Clinical Laboratory Science, Inner Mongolia Medical University, Hohhot, Inner Mongolia, China; ^2^ Department of Clinical Test Center, Medical Laboratory, Peking University Cancer Hospital (Inner Mongolia Campus), Affiliated Cancer Hospital of Inner Mongolia Medical University, Hohhot, Inner Mongolia, China; ^3^ Department of Clinical Test Center, Peking University Cancer Hospital (Inner Mongolia Campus), Affiliated Cancer Hospital of Inner Mongolia Medical University, Hohhot, Inner Mongolia, China

**Keywords:** shikonin, reactive oxygen species, anti-cancer, mechanism, nanomedicine

## Abstract

Excessive buildup of highly reactive molecules can occur due to the generation and dysregulation of reactive oxygen species (ROS) and their associated signaling pathways. ROS have a dual function in cancer development, either leading to DNA mutations that promote the growth and dissemination of cancer cells, or triggering the death of cancer cells. Cancer cells strategically balance their fate by modulating ROS levels, activating pro-cancer signaling pathways, and suppressing antioxidant defenses. Consequently, targeting ROS has emerged as a promising strategy in cancer therapy. Shikonin and its derivatives, along with related drug carriers, can impact several signaling pathways by targeting components involved with oxidative stress to induce processes such as apoptosis, necroptosis, cell cycle arrest, autophagy, as well as modulation of ferroptosis. Moreover, they can increase the responsiveness of drug-resistant cells to chemotherapy drugs, based on the specific characteristics of ROS, as well as the kind and stage of cancer. This research explores the pro-cancer and anti-cancer impacts of ROS, summarize the mechanisms and research achievements of shikonin-targeted ROS in anti-cancer effects and provide suggestions for designing further anti-tumor experiments and undertaking further experimental and practical research.

## 1 Introduction

Aerobic respiration, as an efficient mode of energy production, is the basis for the growth and development of eukaryotic organisms. ROS are highly reactive oxygen-containing molecules generated as byproducts of aerobic respiration and are crucial for the functioning of organisms (Cheung and Vousden, 2022a). ROS, when in the proper concentration and location, are important messengers for cell signaling; Conversely, the strong reactivity of ROS can be harmful to DNA, proteins, and lipids. The organism maintains ROS homeostasis through redox reactions, and when the delicate balance is disturbed in pathological states, the organism undergoes ROS accumulation and oxidative stress (Aggarwal et al., 2019). Therefore, ROS are considered to cause DNA harm and participate in the initiation of cancer (Moldogazieva et al., 2018). It’s been confirmed that many cancer cells display various forms of oxidative stress and elevated concentrations of ROS, at the same time cancer cells boost their antioxidant levels, which makes controlling ROS levels an effective target for cancer treatment (Jelic et al., 2021). Interestingly, ROS suppress cancer by exerting cytotoxic effects, triggering anticancer signaling, and driving cancer cell death through oxidative stress (Perillo et al., 2020). The duality of ROS, together with cancer cells’ heightened susceptibility to ROS and redox processes, suggests that directing treatment towards ROS might be a promising approach in cancer therapy.

Natural products, extracted from various living organisms, exhibit a wide array of unique structures and distinctive biological activities, making them essential in finding and creating new medications. The anticancer potential of these natural entities can be utilised through a comprehensive understanding of their biological attributes. By modifying, optimizing and refining the chemical constituents of these natural molecules, it is feasible to design therapeutic agents that exhibit reduced drug resistance and elicit minimal adverse side effects (Newman and Cragg, 2020). The available data indicates that a significant proportion of approximately 34% of the pharmaceuticals approved by the U.S. Food and Drug Administration (FDA) are derived from natural sources or substances derived therefrom (Atanasov et al., 2021). Shikonin, as documented in Shen Nong’s Herbal Classic, holds a revered position in traditional Chinese medicine. This botanical drug is sourced from the dried roots of Arnebia euchroma (Royle) Johnst or Arnebia guttata Bunge, which are harvested from the regions of Xinjiang and Inner Mongolia. The therapeutic properties of its active component, Shikonin, are multifaceted, encompassing the abilities to clear heat, cool the blood, activate blood circulation, remove toxins, and alleviate rashes, as well as to eliminate dark spots. It is particularly indicated for the treatment of conditions characterized by blood heat and toxicity, such as purple and black spots, stubborn measles, sores, eczema, and various types of burns. The 2020 edition of the Chinese Pharmacopoeia lists Shikonin as a valuable ingredient in the formulation of medicinal products, underscoring its enduring role in the field of healthcare within Chinese culture (Committee NP, 2020). Modern pharmacological research indicates that Shikonin has several therapeutic benefits such as antiviral, antioxidant, anticancer, anti-inflammatory, and bactericidal activities. Notably, the research into the antitumor potential of Shikonin is gaining traction and has emerged as a focal point within the domain of traditional Chinese medicine (TCM) research dedicated to cancer treatment (Guo et al., 2019a). Extensive research indicates that Shikonin can modulate ROS to regulate apoptosis and autophagy, thus, it prevents the dissemination and infiltration of cancer cells, modulates the halting of the cell cycle, and boosts the responsiveness of drug-resistant cells to chemotherapeutic agents. Therefore, the current study investigates the diverse functions of ROS in the progression of cancer, with the aim of generating novel insights that could enhance subsequent research efforts and therapeutic strategies in the field of oncology.

## 2 Sources and control of reactive oxygen species

ROS are formed as oxygen accepts two electrons, resulting in byproducts such as superoxide anion (O^2−^), hydrogen peroxide (H_2_O_2_), hydroxyl radical (OH^−^), as well as lipid, protein, and nucleic acid peroxides (Sies, 2020). They maintain an ongoing equilibrium through oxidation-reduction processes, wherein H_2_O_2_ acts as a signaling molecule that modifies cellular regulatory circuits by selectively changing and controlling the function of certain proteins (Halliwell, 2022). Meanwhile, various types of ROS may potentially cause damage and toxic effects on cells (Hayes et al., 2020).

### 2.1 Sources of ROS

Mitochondria are the main generator of ROS. In mammals, the mitochondrial electron transport chain (ETC) is the primary generator of ATP. During oxidative phosphorylation, around 1% of the molecular oxygen molecules get electrons and escape from the, ETC (Okoye et al., 2023), Superoxide radicals are generated within either Complex I or Complex III of the mitochondrial electron transport chain. Superoxide radicals are released into the cytosol via the mitochondrial permeability transition pore (MPTP) located in the outer mitochondrial membrane (OMM), which is positioned on the external surface of the mitochondrial membrane (Crompton, 1999). Superoxide radicals are mostly transformed into hydrogen peroxide (H_2_O_2_) by superoxide dismutases (SODs), such MnSOD and Cu/ZnSOD (Rhee, 2006). Due to its high diffusibility, H_2_O_2_ is specifically transported into the cytosol by aquaporin channels (specifically aquaporin-3 and aquaporin-8), where it acts as a second messenger to regulate many signaling pathways. Aside from mitochondria, nicotinamide adenine dinucleotide phosphate (NADPH) oxidases (NOXs) are a major source of ROS (Coant et al., 2006). Under normal conditions, The NOX series is triggered by catalytic subunits including Rac1, p47^phox^, p22^phox^, p67^phox^, and 40^phox^, which aid in the transport of electrons to oxygen, leading to the production of O^2−^. Subsequently, this O^2−^ is oxidized to H_2_O_2_ by SOD (Parascandolo and Laukkanen, 2019). These pathways are some known sources of ROS. However, the production of ROS is multifaceted and also includes misfolding of proteins under endoplasmic reticulum stress, cytochrome P450 enzymes, the Fenton reaction involving transition metal ions, and external factors such as tumor necrosis factor-α (TNF-α), interleukin-1β (IL-1β), and epidermal growth factor (EGF). Additionally, hypoxic conditions are also a source of ROS. These examples highlight the complexity of ROS generation and indicate the multiple pathways by which cells may produce ROS in response to a variety of physiological and pathological conditions (Tu and Weissman, 2002).

**FIGURE 1:**
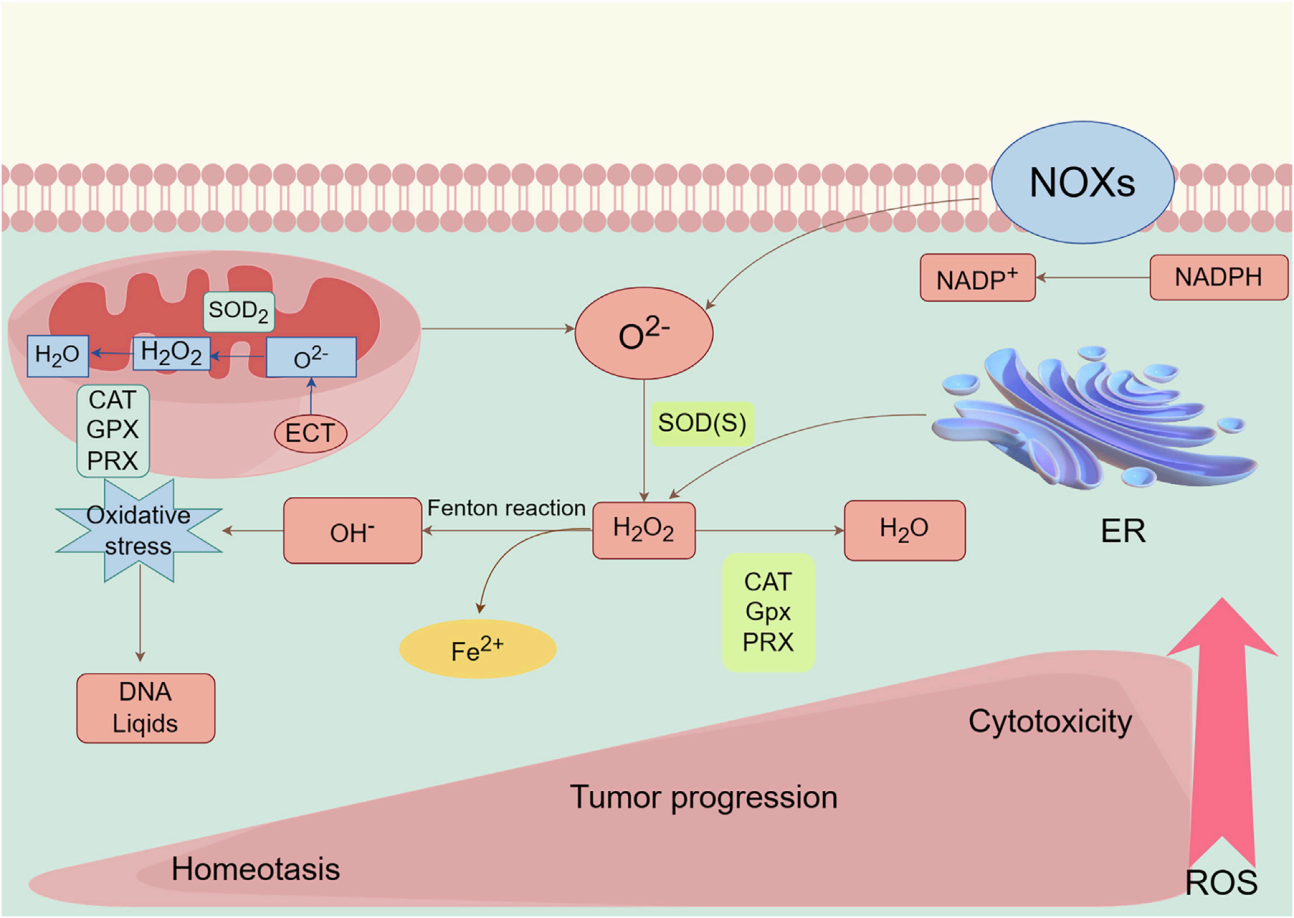
Main generation and modulation of ROS. Mitochondrial and membrane NADPH oxidases (NOXs) are the main sources of endogenous ROS production. The superoxide dismutase (SOD) enzymes transform superoxide radical anion (O^2−^) into hydrogen peroxide (H_2_O_2_). H_2_O_2_ can undergo Fenton chemistry with Fe^2+^ to form a hydroxyl radical (OH^−^), causing damage to DNA, lipids. H_2_O_2_ can be reduced and converted to H_2_O by peroxiredoxins (PRXs), glutathione peroxidases (GPXs), and catalase (CAT). ROS, as a signaling molecule, impacts the development and progression of normal body and disease cells depending on its concentration.

Mitochondria and NOX are two primary sources of ROS, and their spatial localization can be achieved through the action of redox receptors to modulate ROS dependent signaling pathways. H_2_O_2_, with its significant diffusibility, acts as a secondary messenger, controlling various signaling pathways. However, excessive quantities of ROS can cause H_2_O_2_ to move from its original location, resulting in oxidative harm and cell demise. Thus, the advantages and disadvantages of ROS are heavily influenced by the specific regional density and variety of ROS, together with the presence of antioxidants (Wang et al., 2021) (Figure 1).

### 2.2 Modulation of ROS

The antioxidant defense mechanism carefully maintains a delicate equilibrium between ROS generation and their neutralization, thus upholding redox equilibrium and protecting biomolecules from the haphazard destruction brought about by oxidative stress.

#### 2.2.1 Intracellular antioxidant enzymes

Cells deploy a array of antioxidant enzyme systems to regulate ROS, including SOD, which converts O^2−^ into H_2_O_2_. Furthermore, a group of enzymes is involved in the transformation of H_2_O_2_ into water, including catalase (CAT), peroxiredoxin (PRDX), and glutathione peroxidase (GPX) (Rhee, 2016; Nguyen et al., 2020; Fransen and Lismont, 2024), PRDX is a fundamental element of the cell’s antioxidative protective machinery, notable for its high attraction to H_2_O_2_ and its widespread distribution throughout different cellular compartments. After oxidation, PRDX is regenerated by thioredoxin (TRX), which is in turn restored by thioredoxin reductase (TRXR) with the help of NADPH as an electron donor (Gebicka and Krych-Madej, 2019). GPX is pivotal in the conversion of hydrogen peroxide (H_2_O_2_) into water (H_2_O), aiding in its elimination. This process involves the oxidation of reduced glutathione (GSH) into its oxidized form (GSSG). Concurrently, glutathione reductase (GR) utilizes NADPH as a source of electrons to reduce the oxidized GSSG back to its active GSH form. This redox cycle is pivotal for maintaining the cellular glutathione pool, which is essential for protecting cells from the oxidative damage caused by ROS (Barber et al., 2016). In contrast to PRDX and GPX, CAT performs the conversion of hydrogen peroxide (H_2_O_2_) to water (H_2_O) without the requirement of any cofactor. CAT is intricately involved in two distinct antioxidant responses, which are modulated by the concentration of H_2_O_2_. At elevated levels of H_2_O_2_, CAT demonstrates its catalytic prowess by splitting H_2_O_2_ into H_2_O and oxygen (O_2_). Conversely, at lower H_2_O_2_ concentrations, CAT shifts its function to display peroxidase activity by using two reducing equivalents from non-NADPH donors of hydrogen, including metals, hormones, alcohols, and phenols to reduce one molecule of H_2_O_2_ into two molecules of H_2_O. This versatile activity of CAT is vital for regulating H_2_O_2_ levels and safeguarding cells against oxidative damage (Galasso et al., 2021).

#### 2.2.2 NADPH major intracellular hydrogen donor

NADPH is a crucial component of the major intracellular hydrogen donor and reductant pool. The majority of proteins sustain the reduction of moderately reduced disulfide bridges using the NADPH-dependent TRXR and GR redox systems (Smith et al., 2020). NADPH functions as a pivotal electron carrier, simultaneously acting as both a generator of ROS through incomplete reduction and a detoxifying agent for ROS through complete reduction. Ironically, a decrease in NADPH levels can result in the buildup of ROS, while an excess of NADPH can trigger redox imbalance, particularly when NOX enzymes exploit it to generate ROS (Hayes et al., 2020; Sies and Jones, 2020). Accordingly, the antioxidant defense mechanisms of NADPH can only be effectively activated when the levels of NADP+/NADPH are maintained in a state of equilibrium.

#### 2.2.3 Transcription factor

The nuclear factor erythroid 2-related factor 2 (NRF2) is a basic leucine zipper (bZIP) transcription factor with a cap-binding cyclic nuclear localization signal (CNC) structure, which regulates the activation of genes encoding antioxidants to protect cells against oxidative and electrophilic damage (He et al., 2020). The process of ubiquitination, directed by the inhibitory protein Keap1 (Kelch-like ECH-associated protein 1) in conjunction with a CULLIN3-dependent E3 ubiquitin ligase, typically results in the proteasomal degradation of NRF2. Nevertheless, under conditions of ROS stress, the active cysteine residues on KEAP1 are directly altered, which reduces the E3 ubiquitin ligase activity of the KEAP1-CUL3 complex, leading to the stabilization of NRF2 (Suzuki et al., 2023). Upon stabilization, the NRF2 protein is shuttled into the nucleus, where it binds to antioxidant response elements (AREs) and stimulates the transcription of key enzymatic antioxidants, (e.g., CAT, PRX, and GPX), which are indispensable for the preservation of cellular redox homeostasis (Panieri and Saso, 2021). Additionally, NRF2 induces the expression of enzymes crucial for glutathione (GSH) metabolism, notably including heme oxygenase-1 (HO-1) and NAD(P)H: quinone oxidoreductase 1 (NQO-1) (Wang L. et al., 2022). HO-1, a downstream gene of NRF2, plays a fundamental role in the catabolism of heme into biliverdin, iron, and carbon monoxide, the latter of which possesses anti-inflammatory and antiproliferative attributes (Zhang et al., 2021). Similarly, NQO-1, another downstream gene of NRF2, is responsible for the reduction of quinones, toxic intermediates in redox reactions, thereby serving as a critical protective mechanism against oxidative damage (Samarghandian et al., 2020). Furthermore, a diverse set of transcription factors, including the tumor suppressor p53, Activator Protein 1 (AP-1), HIF-1α, Nuclear Factor-κB (NF-κB), and members of the Forkhead Box O (FOXO) family, can be triggered by ROS to regulate the cellular redox equilibrium (Hernansanz-Agustín and Enríquez, 2021). The synergistic actions of NRF2 and these transcription factors underscore the sophistication of the cellular response to oxidative stress and highlight the imperative of maintaining an optimal redox environment for the promotion of cellular health and the prevention of disease.

## 3 The ambivalent function of ROS in cancer

**FIGURE 2:**
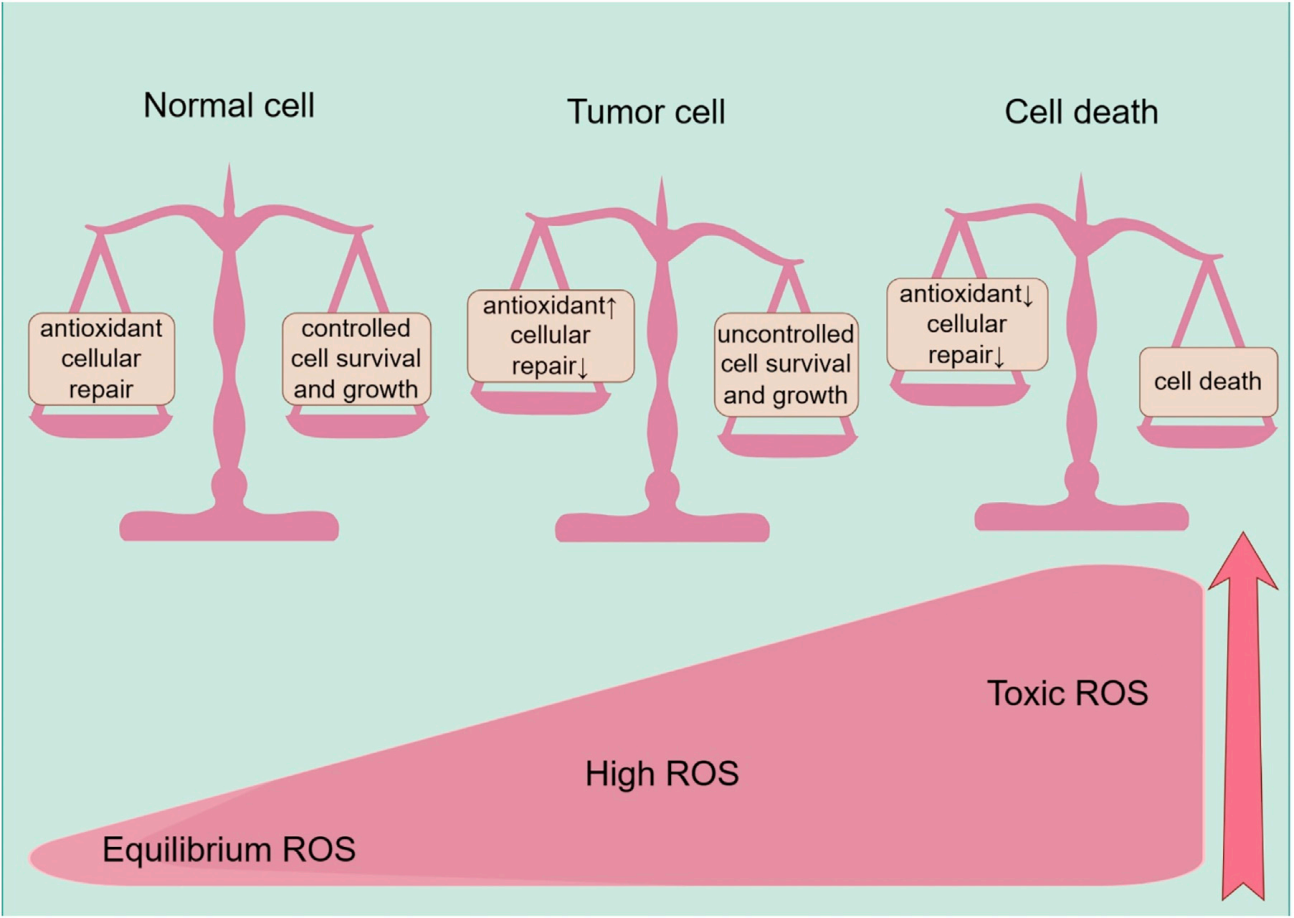
ROS effects on cells, including the normal cells, tumor progression, and cell death. Healthy cells have a well-balanced production of ROS, adequate antioxidant activity, and cellular repair, resulting in appropriate concentrations of ROS that limit cell survival and proliferation. Increased levels of ROS can cause cellular damage, yet tumor cells express the enhanced antioxidant activity and maintain pro-tumor signaling through adequate adaptation to conditions including hypoxia and through metabolic readjustment. However, ROS levels increase to toxic concentrations, and oxidative stress leads to irreparable damage to cells, inadequate adaptation, and, ultimately, tumor cell death.

ROS’s problematic significance in cancer is defined by its dual effects, which are influenced by their varying concentrations. Elevated quantities of ROS have the potential to cause DNA damage and mutations, thus promoting the development and advancement of cancer, while also aiding in the growth and spread of cancer. However, excessive ROS levels can induce RCD if they exceed a certain threshold, known as apoptosis, autophagy, ferroptosis, or necroptosis, thus demonstrating antitumor effects. The net effect of ROS on cancer—whether they function as a carcinogen or a tumor suppressor—is determined by their dynamic concentrations, distribution, and duration, resulting in distinct roles throughout the cancerous journey (Wang et al., 2021) (Figure 2).

### 3.1 The carcinogenic effects of ROS

ROS has a complex function in facilitating the growth of tumors and the advancement of tumors (Cheung and Vousden, 2022b). Prolonged exposure to elevated levels of ROS can result in DNA impairment and heightened genomic instability, serving as key drivers for the onset of cancer (Srinivas et al., 2019). Additionally, ROS can impact signaling pathways by reversibly oxidizing proteins crucial for cancer cell functions such as survival, proliferation, metabolism, invasion, and metastasis (Wang et al., 2021).

#### 3.1.1 ROS driven cellular proliferation

The engagement of ROS in cancer cell proliferation has been a subject of extensive research. A notable breakthrough has been the recognition of ROS as secondary messengers that participate in the activation of growth factors, mediating this process through the PI3K/AKT/mTOR and MAPK/ERK mitotic signaling pathways. The oxidative damage causing the inactivation of phosphatase and tensin homolog (PTEN) and protein tyrosine phosphatase PT1B compromises their inhibitory effects on the PI3K/AKT/mTOR pathway, thereby affecting cell proliferation (Lee et al., 2002). The pivotal role of PI3K/AKT in the mitotic signaling cascade renders the upregulation of this channel, caused by the oxidative inactivation of PTEN/PTP1B upstream, a defining feature of malignant tumor (Hu et al., 2019). This is corroborated by the inactivation of PTEN in diverse cancers and the subsequent activation of the ROS-induced PI3K signaling pathway. Neuroblastoma involves the excessive creation of ROS by NADPH oxidases (NOXs) following insulin stimulation. This results in the inhibition of the oncogene PTEN through oxidation and the activation of PI3K/AKT through phosphorylation (Guo et al., 2020). Conversely, in breast cancer cells, the chemokine CXCL12 triggers NOX2, resulting in a transient accumulation of H_2_O_2_. This leads to the oxidative inactivation of PTEN and PTP1B, stimulation of the PI3K/Akt signaling pathway, and maintenance of cyclin D expression, ultimately stimulating cell proliferation (Tong et al., 2019).

The mitogen-activated protein kinase (MAPK) group consists of four key proteins: extracellular signal-regulated kinases 1 and 2 (Erk1/2), c-Jun N-terminal kinase (JNK), p38 mitogen-activated protein kinase (p38), and BMK1/big mitogen-activated protein kinase 1 (BMK1/Erk5). Their activation is managed by a structured kinase sequence that involves three tiers: MAPK kinase kinases (MAPKKKs), MAPK kinases (MAPKKs), and the MAPKs at the end of the cascade (Peluso et al., 2017). Cell apoptosis signal-regulating kinase 1 (ASK1), a member of the mitogen-activated protein kinase kinase kinase (MAPKKK) family, sees its activity suppressed when it binds to the oxidized form of thioredoxin (TRX) (Saitoh et al., 1998). The build-up of ROS or a lack of antioxidants triggers the oxidation of TRX, which results in its separation from ASK1. This event, in turn, leads to the reactivation of ASK1’s kinase activity (Liu et al., 2000). In addition to regulating the upstream factors of MAPKs, ROS can also stimulate MAPKs by directly inhibiting MAPK dephosphorylation enzymes. Research has shown that ROS sustain the activation of JNK by converting the catalytic cysteine to a sulfenic acid, which inhibits the dephosphorylation of JNK and forestalls its inactivation (Kamata et al., 2005).

#### 3.1.2 ROS drive cancer cell metastasis

As malignancies evolve, they acquire the propensity to infiltrate neighboring tissues and to disseminate to distant locations, constituting the primary reason for death in most individuals with cancer. Beyond the fundamental requirements of cell proliferation and survival at the site of origin, the accomplishment of metastasis necessitates an array of additional capabilities, including the propensity to migrate and invade, the resilience to survive the dissolution of normal cellular interactions and perfusion, and the aptitude to penetrate and regenerate within the novel milieu of a distant organ (Chaffer and Weinberg, 2011). ROS facilitates cancer metastasis by activating matrix metalloproteinase (MMP). Degradation of extracellular matrix (ECM) proteins that is reliant on a specific factor, leading to an increase in hypoxia. Hypoxia-inducible factor (HIF) Angiogenesis that relies on a certain factor or condition (Chatterjee and Chatterjee, 2020).

**FIGURE 3:**
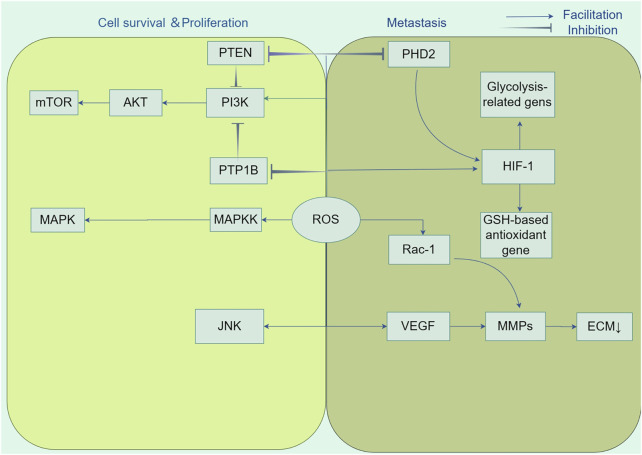
Tumorigenic role of ROS. Reactive oxygen species (ROS) participate in the activation of growth factors as second messengers and promote cancer cell growth and survival by affecting key regulators of multiple signalling pathways, including PI3K/AKT/mTOR and MAPK/ERK, in cancer cell proliferation. In addition, ROS enhanced cancer cell migration and invasion by inhibiting prolyl hydroxylase structural domain protein (PHD2) in the hypoxia signalling pathway and stabilising hypoxia-inducible factor-1α (HIF-1α) subunits. HIF-1 orchestrates signalling cascades to accomplish growth factor-mediated angiogenesis, such as vascular endothelial growth factor (VEGF), by up-regulating the expression of lactate dehydrogenase and pyruvate dehydrogenase kinase 1, down-regulating the expression of glutathione (GSH)-based antioxidant genes, and decreasing the production of mitochondrial ROS. Also, ROS accelerate angiogenesis and promote metastasis by stimulating matrix metalloproteinase (MMP)-dependent degradation of extracellular matrix (ECM) proteins.

Hypoxia-inducible factors (HIFs) are composed of two subunits, HIF-1α and HIF-1β, which serve as key transcriptional regulators that are generally active, orchestrating signal transduction pathways to facilitate angiogenesis (Zhao Y. et al., 2023). ROS promote the movement and penetration of cancer cells by supporting the stability of the oxygen-sensitive HIF-α subunit. This is achieved by blocking the activity of prolyl hydroxylase (PHD2) in the hypoxic signaling pathway (Harris, 2002). HIFs promote the growth of tumor blood vessels by inducing overexpression of the gene encoding vascular endothelial growth factor (VEGF) (Catrina and Zheng, 2021). Moreover, HIF-1 mitigates energy shortages by upregulating the expression of enzymes associated with glycolysis, including lactate dehydrogenase and pyruvate dehydrogenase, thereby elevating the levels of glycolytic metabolism. Additionally, it augments the expression of antioxidant genes that are dependent on glutathione (GSH) to counteract oxidative stress (Stegen et al., 2016). Another feature of many aggressive cancers is the process of epithelial-mesenchymal transition (EMT), which is characterized as an early occurrence in the spread of cancer to other parts of the body. The condition is defined by the absence of cell polarity and separation from adjacent cells, which is regulated by the function of tiny Rho family GTPases (Etienne-Manneville and Hall, 2002). Moldovan et al. have demonstrated that the actin cytoskeleton remodeling induced by RAC1 in human endothelial cells requires the production of superoxide anions (Moldovan et al., 1999). The utilization of the antioxidant N-acetyl-l-cysteine (NAC) to cancer cells has demonstrated the capacity to elicit a reversion to an epithelial phenotype, thereby underscoring the critical involvement of ROS in the EMT process (Li et al., 2020a) (Figure 3).

### 3.2 ROS induce regulated death in cancer cells

When ROS accumulation exceeds a certain threshold, these molecules shift their functional role from promoting carcinogenic processes such as cell proliferation and invasion to exerting anticancer effects by triggering regulated cell death (RCD) pathways. Such as apoptosis, autophagy, ferroptosis, necroptosis, and Oxeiptosis (Hengartner, 2000).

Apoptosis, or type I programmed cell death, is an environmentally triggered, gene-regulated, active, and orderly process of cellular demise (Kerr et al., 1972). Currently, it consists of three main pathways: The mitochondrial pathway (endogenous apoptotic pathway), death receptor pathway (exogenous apoptotic pathway), and endoplasmic reticulum pathway are carried out by cysteine proteases (Oppenheim et al., 2001). Extrinsic apoptosis is initiated by ligand-receptor engagement between death ligands (such as FasL) and tumor necrosis factor (TNF) family members with their specific receptors, including the Fas receptor (FasR) and the tumor necrosis factor receptor (TNFR) (Meynier and Rieux-Laucat, 2019; Pan et al., 2022). Upon ligand-receptor engagement, apoptotic signals prompt the assembly of death-inducing signaling complexes (DISCs) containing Death Domain (DD), Death Effector Domain (DED), FADD (TRADD), and pro-caspase-8 (Medina et al., 2020). Caspase-8 triggers a caspase cascade to license apoptosis, while the death receptor-binding protein c-FLIP blocks this process by competitively engaging procaspase-8 and preventing DISC assembly. ROS promote extrinsic apoptosis by speeding up the degradation of c-FLIP via ubiquitination. NAC treatment stabilizes c-FLIP, facilitating apoptosis, indicating that ROS function as pro-apoptotic agents (Hwang et al., 2021). ROS potentiate the intrinsic apoptosis pathway by pressurizing the mitochondrial permeability transition pore (MPTP), resulting in a reduction of the mitochondrial transmembrane potential and eventual disruption of membrane integrity. This action allows pro-apoptotic substances, such cytochrome c (Cyt-c), to be released into the cytoplasm (Cain et al., 2002). Once released into the cytosol, Cytochrome c links to Apoptotic Protease-Activating Factor 1 (APAF-1) and procaspase-9 to create an apoptosome, which triggers the activation of caspase-9, commencing a signaling cascade that culminates in DNA damage and cell death (Kagan et al., 2005). Apoptosis proteins are central to the intrinsic apoptotic pathway, with ROS modulating Bcl-2 family members to increase mitochondrial outer membrane permeability, resulting in the liberation of Cyt-c (Kalkavan et al., 2022). Furthermore, ROS can modulate relevant signaling pathways, ultimately resulting in apoptosis. JNK and P38 are subfamilies of the MAPK signaling pathway. Tyrosine kinase inhibitors, such as Imatinib (PDGFR inhibitor) and Erlotinib (EGFR inhibitor), activate JNK and P38 through phosphorylation, respectively. Initiating this activation triggers a series of intracellular processes that result in the disruption of mitochondrial membrane potential. The altered mitochondrial function subsequently increases ROS production, which is critical for apoptosis induction in melanoma and non-small cell lung cancer cells (Liu X. et al., 2019; Mihajlovic et al., 2020)

Autophagy is a complex molecular pathway that transports intracellular components to lysosomes for decomposition and reutilization (Debnath et al., 2023). Three main forms of autophagy have been largely described thus far: macroautophagy, selective autophagy, and microautophagy (Rakesh et al., 2022). Macroautophagy involves the encapsulation of cytoplasmic components within double-membrane structures known as autophagosomes (Pan et al., 2019). Bulk autophagy, also known as macroautophagy, involves the non-selective engulfment of cytoplasmic materials into autophagosomes. In contrast, selective autophagy, or chaperone-mediated autophagy (CMA), is a highly selective process that utilizes the chaperone Hsc70 to target proteins with a specific KFERQ-like sequence for degradation. LAMP2A, a lysosomal receptor, recognizes and binds to the exposed KFERQ motif, contributing in transporting these proteins to the lysosome for hydrolysis (Gohel et al., 2020; Bourdenx et al., 2021); Microautophagy is a particular kind of autophagy where cellular components are directly taken in via the creation of membrane-enclosed invaginations on late endosomes or lysosomes. It is initiated by specific signal molecules on impaired organelles, such as mitochondria or peroxisomes, resulting in the selective fusion of lysosomes with these structures (Kuchitsu and Taguchi, 2023; Wang et al., 2023). Extensive evidence indicates that autophagy is directly mediated and regulated by ROS levels in malignant tumours, and ROS can inhibit ATG4 cysteine protease activity, thus supporting an increase in LC3-associated autophagy calibrators (Perillo et al., 2020), NRF2 is a crucial transcription factor that aids cells in countering oxidative stress by governing the expression of genes encoding antioxidants. An excessive production of ROS can trigger the NRF2-p62 pathway, thereby enhancing autophagy—a cellular process that diminishes oxidative injury (Su et al., 2021). Oxidative stress not only activates NRF2 but also the transcription factor forkhead box O3 (FOXO3). Specifically, NRF2 promotes the transcription of p62, while FOXO3 stimulates the expression of LC3 and BNIP3—genes that play pivotal roles in the autophagy-lysosome pathway (Hao et al., 2022). mTORC1 functions as a negative modulator of autophagy, its activity being suppressed by AMPK activation, thereby promoting the initiation of autophagy (Moloney and Cotter, 2018). Glioma cells treatment with hydrogen peroxide (H_2_O_2_) following the administration of cyclamates, such as hesperetin, induces autophagy (Pervaiz et al., 2020).

Necroptosis, referred to as programmed necrosis, is a type of cell death triggered by the interaction of death receptors with their corresponding ligands, genotoxic stress, or specific anti-neoplastic drugs (such as etoposide). It is mediated by the kinases RIP1 and RIP3 (Yuan and Ofengeim, 2024), leading to a type of necrotic cell death that involves increased lysosomal and plasma membrane permeability. Necroptosis is distinct from apoptosis, absence of chromatin condensation, cytoplasmic shrinkage, or nuclear fragmentation, features distinct morphology, and elicits an inflammatory response (Gong et al., 2019; Wang H. et al., 2022). Necroptosis and reactive oxygen species (ROS) create a beneficial cycle that enables their mutual stimulation. ROS generated outside of the mitochondria by NOX1 and ROS produced inside the mitochondria can initiate RIP1 activation and recruitment of RIP3, resulting in necroptosis (Qiu et al., 2018). However, RIP3 can increase aerobic respiration and boost ROS generation via influencing metabolic pathways such as PDH, PYGL and GLUD1 (Wang et al., 2018). Recent studies indicate a strong association between ROS and necroptosis, suggesting that ROS modulation may serve as a promising anti-cancer treatment strategy. Thus, leveraging necroptosis and ROS-mediated cell death could represent a viable approach for cancer cell elimination.

**FIGURE 4:**
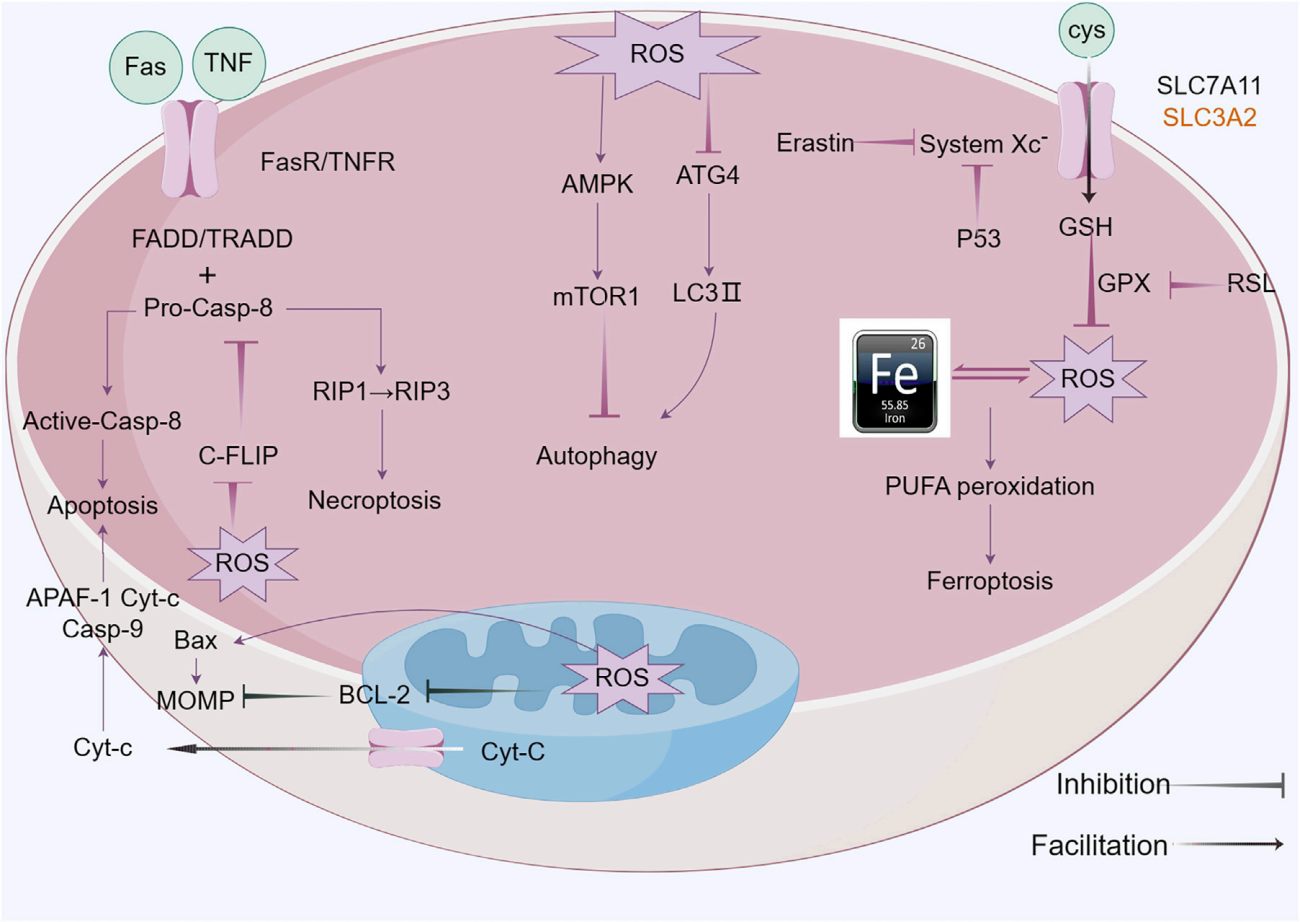
ROS functions of antitumor effects via regulated cell death (RCD) consisting largely of apoptosis, autophagy, necroptosis and ferroptosis. ROS exert pressure on the open mitochondrial permeability transition pore (MPTP), leading to a decreased linear mitochondrial membrane potential (MMP), allowing cytochrome C (Cyt-c) to be released into the cytoplasm and form an apoptotic complex with apoptotic protease activating factor-1 (APAF-1) and procaspase-9 while triggering a caspase-9 signaling cascade that ultimately triggers apoptosis. ROS-dependent proteasomal degradation of cellular FADD-like IL-1β-converting enzyme (FLICE)-inhibitory protein (c-FLIP) enhances the exogenous apoptotic pathway, which is triggered by the splicing of death-inducing ligands with their cognate receptors (FasR). ROS can initiate RIP1 activation and RIP3 recruitment, leading to necroptosis. ROS-dependent inactivation of autophagy-related gene 4 (Atg4) leads to an increase in microtubule-associated protein 1 light chain 3 (LC3)-associated autophagosomes and induces autophagy. mTORC1, a negative regulator of autophagy, is activated by adenosine 5’-monophosphate (AMP)-activated protein kinase (AMPK) and inhibits autophagy. Ferroptosis is caused by ROS-induced iron-dependent lipid peroxidation of PCD. Fenton chemistry increases lipoxygenase activity/ROS production. Erastin impairs the GSH-dependent glutathione peroxidase (GPX) antioxidant system through the mediation of the cystine/glutamate reverse transporter protein (XC− system). Increasing ROS causes changes in the permeability of the outer mitochondrial membrane, and (1S,3R)-RSL3 (RSL3) causes changes in the outer mitochondrial membrane by blocking GPX that stimulates ferroptosis.

Ferroptosis was initially characterized in 2012 by Dixon and colleagues (Dixon et al., 2012), is an iron-dependent form of regulated cell death (RCD) that is marked by the build-up of lipid reactive oxygen species (ROS) (Jiang et al., 2021), which exhibits distinct morphological, biochemical, and genetic characteristics, differentiating it from both apoptosis and necrosis (Tang et al., 2021). The characteristics of ferroptosis are excessive intracellular iron levels as well as oxidative stress due to insufficient glutathione (GSH), resulting in deadly buildup of oxidized polyunsaturated fatty acids (PUFAs) (Li et al., 2020b; Tang et al., 2021). ACSL4 and LPCAT3, respectively members of the long-chain acyl-CoA synthetase family and the lysophosphatidylcholine acyltransferase family, act as key executors of ferroptosis. ACSL4 catalyzes the attachment of long-chain PUFAs (including arachidonic acid and adrenic acid) to CoA, a process that is followed by LPCAT3-mediated re-esterification within phospholipids (Zou et al., 2020). Additionally, Lipoxygenases (LOXs) and cytochrome P450 reductase (POR) play roles in the process of lipid peroxidation (Borsini et al., 2021). The system xc- (cysteine/glutamate exchanger) and GPX4 are pivotal cellular factors that, when inhibited by the compounds erastin and RSL3, respectively, contribute to the onset of ferroptosis (Djulbegovic and Uversky, 2019; Jang et al., 2021). System XC- is a heterodimeric transmembrane protein consisted of SLC7A11 and SLC3A2, which is crucial for preserving the balance of redox processes by aiding in the transportation of cysteine necessary for glutathione (GSH) production. Research indicates that p53, a tumor suppressor protein, inhibits cysteine acquisition by inhibiting SLC7A11 expression and inducing ferroptosis (Koppula et al., 2021). Iron is a crucial component in the execution of ferroptosis (Galy et al., 2024), serving as a requirement for the catalytic activity of metabolic enzymes, such as lipoxygenases (LOXs) and paraoxonase (POR), which are involved in phospholipid peroxidation; Intracellular Fe^2^+ reacts with H_2_O_2_ through Fenton’s reaction, generating ROS that initiate ferroptosis (Conrad and Pratt, 2020). Conversely, iron chelation can prevent this form of cell death. Deferoxamine (DFO), a non-permeable iron chelator, is taken up by cells via endocytosis and accumulated in lysosomes, where it intercepts iron ions destined for other cellular locations, effectively inhibiting the production of lipid ROS and thereby preventing ferroptosis (Barradas et al., 1989) (Figure 4).

**FIGURE 5:**
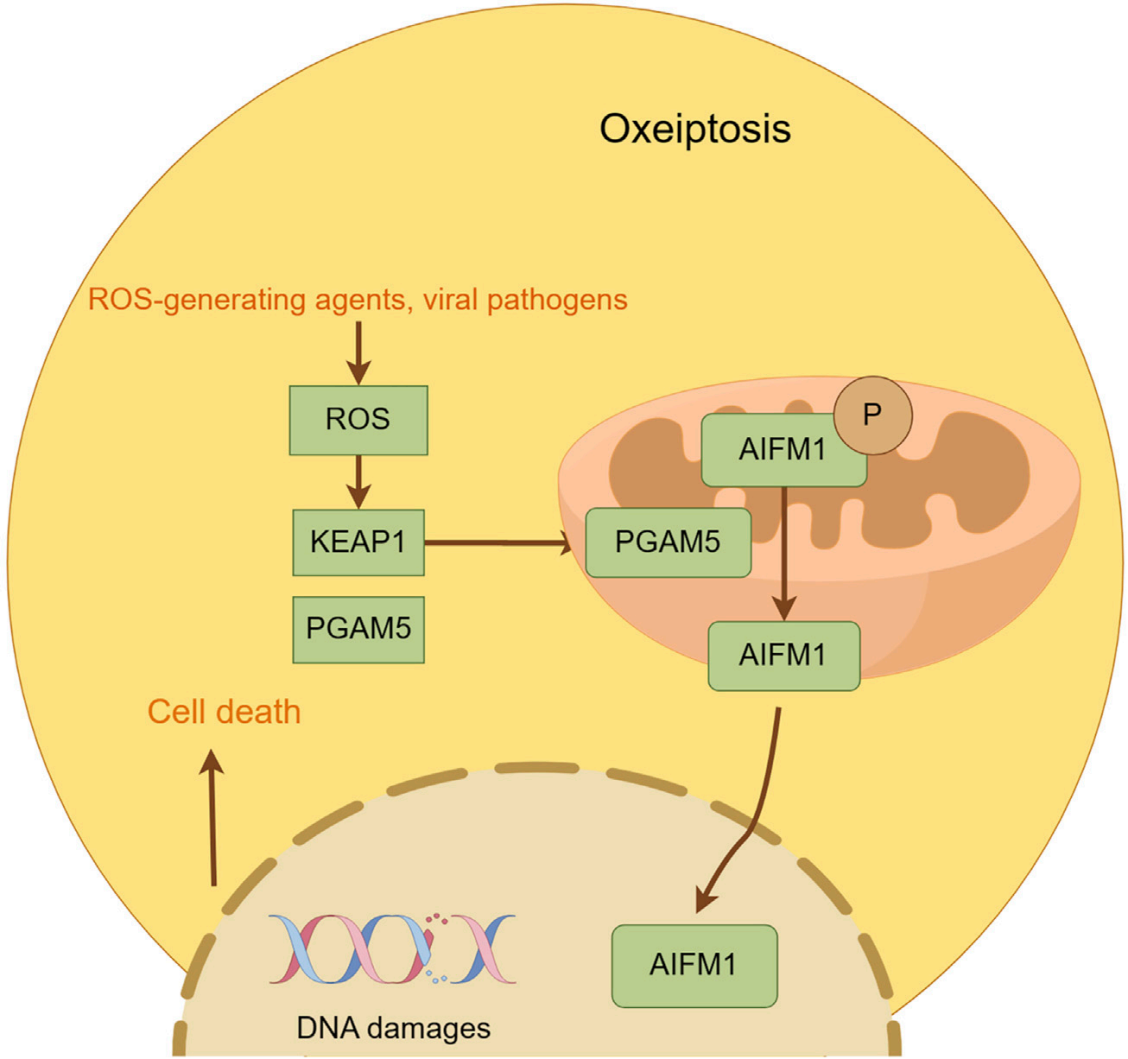
This figure illustrates the key features of oxeiptosis. Oxeiptosis is activated in response to oxidative stress induced by ROS or ROS-generating agents, such as viral pathogens. The KEAP1/PGAM5/AIFM1 signaling pathway plays a central role in oxeiptosis, in which AIFM1 is dephosphorylated under oxidative stress conditions via the regulatory action of PGAM5. Dephosphorylated AIFM1 is translocated from mitochon-dria to the nucleus, leading to chromatin condensation and DNA fragmentation, ultimately resulting in cell death.

Oxeiptosis is a novel cell death mechanism independent of caspases and driven by ROS. First described by Holze et al. (2018) in 2018, this process was observed in fibroblasts exposed to ozone and hydrogen peroxide. It is regulated by a signaling cascade involving KEAP1, PGAM5, and AIFM1. KEAP1, an oxidative stress sensor, is crucial for the activation of oxeiptosis by ROS(95). KEAP1 can either retain Nrf2 in the cytoplasm and degrade it through ubiquitination or release it in response to ROS, facilitating its nuclear translocation (Kobayashi et al., 2004). Once in the nucleus, Nrf2 binds to AREs and promotes the expression of antioxidant genes, protecting cells from ROS damage (Otsuki and Yamamoto, 2020). High levels of ROS, particularly hydrogen peroxide, can overactivate KEAP1, initiating the oxeiptosis pathway through PGAM5. PGAM5, released from KEAP1, enters the mitochondria and induces dephosphorylation of AIFM1, leading to the activation of oxeiptosis and cell death (Zeb et al., 2022). AIFM1 is a key player in the oxeiptosis signaling pathway, functioning within the mitochondria rather than being translocated to other cellular compartments. When triggered by PGAM5, AIFM1 undergoes dephosphorylation at Serine 116, activating oxeiptosis (Holze et al., 2018). AIFM1 has been identified as a mutant form of AIF in various disease contexts, impacting cellular processes such as apoptosis and auditory nerve function. Research has linked oxeiptosis to a variety of diseases. For example, the upregulation of Keap1 and AIFM1 by Auriculasin has been shown to enhance ROS production and inhibit tumor cell growth in colorectal cancer cells. Conversely, the inhibition of KEAP1 and AIFM1 can reduce this inhibitory effect (Wang C-x et al., 2022). Additionally, ROS, especially hydrogen peroxide, has been suggested as a potential treatment for colon cancer by inducing oxeiptosis, a process that can be reversed by ROS scavengers (Pallichankandy et al., 2023). In cutaneous melanocytes under oxidative stress, AIFM1 dephosphorylation at Serine 116 has been associated with the induction of melanocyte death, or oxeiptosis, which can lead to conditions like vitiligo (Kang et al., 2022) (Figure 5).

## 4 Shikonin’s anti-cancer mechanism targeting ROS

### 4.1 Overview of shikonin

Shikonin, a prominent bioactive compound extracted from the roots of Lithospermum erythrorhizon, commonly referred to as “Zicao” in TCM. In traditional medical practices, shikonin is commonly employed for its anti-inflammatory and antiseptic properties, as well as for its abilities to counteract heat and toxicity, and to promote the acceleration of wound healing processes. Multiple studies have shown that shikonin exhibits anti-inflammatory activities in various models of acute inflammation and injury. For example, the anti-inflammatory activity of shikonin was verified by Yang et al., shikonin was found to mitigate the rise in ALT and AST levels triggered by LPS/GalN, improved histological abnormalities by suppressing TLR4 expression and NF-κB activation, and reduced the secretion of TNF-α and IL-1β (Yang et al., 2017). Lu et al. demonstrated the anti-inflammatory properties of shikonin within two traditional mouse models frequently employed in pharmacological research to assess anti-inflammatory activities: the xylene-induced ear edema model and the acetic acid-induced capillary permeability model (Lu et al., 2011). In the mouse model of cerulein-induced acute pancreatitis, shikonin demonstrated a significant protective effect against histological injury. This is evidenced by reduced pancreatic pathology scores, decreased activity of myeloperoxidase (MPO), and lower serum levels of amylase and lipase. The underlying mechanisms are related to the inhibition of the NF-κB signaling pathway and a decrease in the release of pro-inflammatory cytokines, including TNF-α, IL-1β, and IL-6 (102). However, Based on Yan et al. (2015)’s research, shikonin appears to enhance wound repair by encouraging the proliferation of healthy human keratinocytes and human dermal fibroblasts. Epithelial-mesenchymal transition (EMT) plays a crucial role in wound healing. Shikonin stimulates EMT by weakening the nuclear translocation of NF-κB p65 in human dermal fibroblasts and enhancing the expression of specific EMT regulatory molecules, including the transcription repressors of E-cadherin, ZEB1 and ZEB2, which effectively promotes the wound healing process of damaged skin tissue (Yin et al., 2013).

**FIGURE 6:**
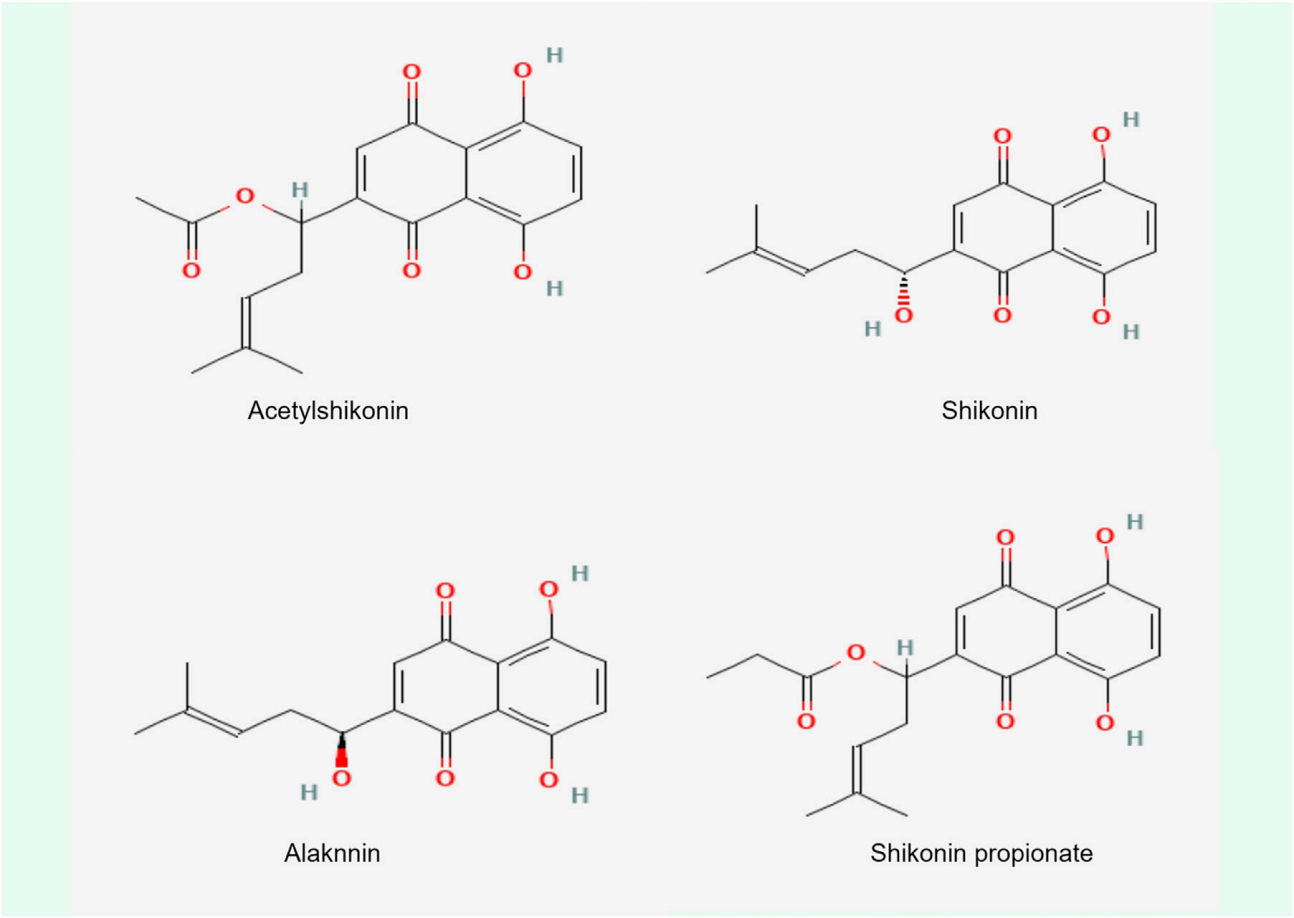
Chemical structure of four shikonin types.

**FIGURE 7:**
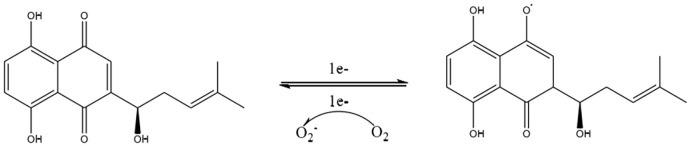
Electron transfer mechanism of naphthoquinone.

Shikonin, a complex compound that is rich in a diversity of chemical metabolites such as naphthoquinones, flavonoids, phenolic acids, terpenoids, and polysaccharides, possesses notable antitumorigenic properties. These properties are primarily associated with the unique structural characteristics of its α-1,4 naphthoquinone parent moiety (Figure 6). The naphthoquinone framework possesses the capacity to produce ROS, which in turn modulate cellular oxidative stress levels (Adjobo-Hermans et al., 2020). The mechanism by which this occurs is detailed (Figure 7). It involves the semiquinone residue of the naphthoquinone ring undergoing a uniprotocol electron transfer reduction reaction, followed by re-oxidation to the quinone state in the presence of oxygen molecules. This redox shuttle activity results in the production of substantial quantities of ROS. The elevated levels of ROS can then inflict damage on cellular proteins, lipids, and DNA, which consequently impacts the membrane potentials of both the cellular and mitochondrial compartments (Chen et al., 2002; Kumar et al., 2021; Yadav et al., 2022; Zhao M. et al., 2023). Accordingly, the oxidative-reductive capabilities of the naphthoquinone ring are utilized, with an emphasis on the anticancer impacts of shikonin mediated by the production of high levels of ROS.

The therapeutic potential of Shikonin against a range of systemic malignancies has been thoroughly investigated, encompassing lung cancer, lymphoma, leukemia, gastric cancer, colorectal cancer, hepatocellular carcinoma, pancreatic cancer, and breast cancer (Tang et al., 2018; Guo et al., 2019b; Ji et al., 2022; Li et al., 2022; Xu et al., 2022; Dong et al., 2023; Király et al., 2023; Qian et al., 2023; Zhang et al., 2023). Shikonin is rich in various chemical constituents, including naphthoquinones, flavonoids, phenolic acids, terpenes, and polysaccharides.

Research has demonstrated that Shikonin possesses antitumor capabilities by modulating a plethora of pathways, such as triggering apoptosis, autophagy, necroptosis, ferroptosis, halting the cell cycle, overcoming drug resistance, and potentiating the impact of chemotherapy, with all these effects being mediated through the control of ROS (Zhu et al., 2012; Zhang et al., 2018). The upcoming section explores the anticancer mechanisms involving the targeting of ROS by individual components metabolites in Shikonin.

### 4.2 The anti-cancer mechanism of shikonin targeting ROS

#### 4.2.1 Shikonin induces apoptosis in cancer cells by targeting ROS

Elevated levels of ROS can trigger cell death. The regulatory role of Shikonin in the apoptosis induced by ROS, which encompasses both endogenous and exogenous apoptotic pathways, is increasingly esteemed in cancer treatment strategies. Additionally, the predominant function of ROS in selectively acting on the ER or energy metabolism pathways to provoke apoptosis in a diverse array of cancer cell lines has been emphasized by many investigators. Shikonin is fundamental in facilitating the apoptotic processes mentioned above.

A considerable amount of research has elucidated that shikonin induces mitochondria-mediated apoptosis in cancer cells through a ROS-dependent mechanism. Studies focusing on primary effusion lymphoma (PEL) have shown that ROS are the proximal initiating factors for shikonin to trigger caspase-dependent apoptosis in PEL cells. Excessive ROS can cause damage to the outer mitochondrial membrane, leading to membrane depolarization and activation of the intrinsic apoptotic pathway. Concurrently, ROS also stimulate the extrinsic JNK signaling pathway. As a ROS scavenger, the use of NAC can almost eliminate the loss of mitochondrial membrane potential (Δψm) and prevent apoptosis in these cells. In addition, researchers have established a PEL cell (human GTO cells) growth model in nude mice. The experimental results showed that compared to the control group treated with PBS alone, the growth of PEL cells in nude mice treated with shikonin was significantly inhibited, as evidenced by: 1) changes in volume and weight, 2) changes in spleen weight, and 3) changes in ascites volume. These results demonstrate that shikonin has a significant inhibitory effect on the proliferation of PEL cells in the mouse model, providing strong *in vivo* experimental evidence for the treatment of PEL with shikonin (Alam et al., 2021). Further studies on shikonin’s effects against adult T-cell leukemia/lymphoma (ATLL) reinforce the notion that ROS is a key initiator of apoptosis in ATLL cells mediated by shikonin, via disruption of mitochondrial membrane potential and ER stress (Boonnate et al., 2023). Shikonin reduces the functionality of antioxidant enzymes, thereby promoting the onset of intrinsic apoptosis. Thioredoxin reductase 60 (TrxR60), a crucial antioxidant enzyme with selenocysteine (Sec), is a primary target of shikonin. Duan and colleagues demonstrated that shikonin specifically inhibits the physiological function of TrxR1 by targeting its Sec residue. This inhibition alters the enzyme’s function to act as a NADPH oxidase, producing superoxide anions. Consequently, this mechanism results in the accumulation of ROS and disrupts the intracellular redox homeostasis. In addition, using a vector-transfected cell line as a control for TrxR overexpression and knockdown experiments, it was found that TrxR1 overexpression reduced the cytotoxicity of shikonin, while TrxR1 knockdown increased its cytotoxicity after treatment with different concentrations of shikonin acid (1–1.5 μM) (Duan et al., 2014). ER stress-induced apoptosis is a key pathway, and shikonin, derived from Lithospermum, targets ROS via this route. Qi’s team investigated shikonin’s inhibitory effects on colorectal cancer cells using *in vitro* and *in vivo* models. Western blot analysis revealed that shikonin treatment enhanced phosphorylation of PERK, eIF2α, ATF4, IRE1α, and JNK, indicating ER stress activation. *In vivo*, a xenograft model was created by injecting HCT-116 and HCT-15 cells into BALB/c nude mice. *In vivo* Western blot of HCT-15 xenografts showed shikonin upregulated PERK/eIF2α/ATF4/CHOP and IRE1α/JNK pathways. While oral shikonin did not demonstrate anti-tumor effects in the colorectal cancer model, intraperitoneal injection significantly inhibited tumor growth. The antitumor effects of intraperitoneal shikonin were more pronounced. However, pharmacokinetic (PK) analysis is needed to understand the differences in exposure and metabolite profiles between oral and intraperitoneal administration routes (Qi et al., 2022). Shikonin employs extrinsic apoptosis as a mechanism to curtail cancer cell proliferation by modulating ROS. Zhou G. et al. (2017) have shown that shikonin augments the manifestation or upregulation of Death Receptor 5 (DR5) in cholangiocarcinoma cells through ROS-induced activation of the JNK signaling cascade. This activation triggers a cascade leading to the activation of downstream caspase proteases, such as Caspase-3, Caspase-8, and Caspase-9, thereby promoting apoptosis mediated by TRAIL (Tumor Necrosis Factor-Related Apoptosis-Inducing Ligand) treated cholangiocarcinoma cells. In the pursuit of innovative strategies for cancer therapy, targeting glycolytic enzymes has emerged as a pivotal approach. Dr. Wei’s research team employed two human hepatocellular carcinoma cell lines, HepG2 and HCCLM3, as *in vitro* models to investigate the effects of treatment with zwitterionin. Two control groups were established: one with untreated cells serving as a baseline control, and another with NAC treated cells as a positive control for ROS inhibition. Their findings indicated that zwitterionin inhibited glycolysis in HepG2 cells by suppressing the activity of PKM2, a critical enzyme within the glycolytic pathway. Remarkably, HepG2 cells demonstrated heightened sensitivity to viologen-induced apoptosis compared to HCCLM3 cells. This differential response may be attributed to several factors: 1) Difference in ATP levels: HCCLM3 cells exhibited a substantially higher basal ATP level than HepG2 cells. This difference in energy metabolism may represent one of the mechanisms underlying the resistance to apoptosis observed in HCCLM3 cells; 2) Variation in PKM2 and HIF-1α expression: HCCLM3 cells expressed PKM2 and HIF-1α at higher levels. The upregulation of these proteins may suppress mitochondrial biogenesis and promote glycolysis, thereby diminishing the susceptibility of HCCLM3 cells to the effects of zwitterionin. This research highlights the potential for targeting glycolytic enzymes in the treatment of hepatocellular carcinoma and underscores the importance of considering cellular metabolic differences when developing therapeutic strategies (Yang et al., 2021). MAPKs constitute a group of serine/threonine protein kinases that are widely expressed in eukaryotic cells. The MAPK signaling cascade serves to activate the unfolded protein response (UPR), a cellular mechanism that safeguards cells from ER stress-induced damage (Read and Schröder, 2021). Shan and colleagues conducted *in vitro* experiments and found that after using shikonin, the expression levels of p-ERK and c-Myc decreased, while the expression levels of p-p38, MAPK, and p-JNK increased. These findings suggest that shikonin may regulate the proliferation of tumor cells by targeting the MAPK pathway and c-Myc. However, this study did not consider animal model experiments or clinical trials, so the research scope is limited to the mechanism of action and *in vitro* effects of shikonin on NB4 cells. Due to the lack of *in vivo* validation, follow-up studies are needed to further explore the role of shikonin *in vivo*. The ultimate goal is to develop a new therapy for the treatment of acute promyelocytic leukemia (APL) (Shan et al., 2017).

#### 4.2.2 Shikonin induces autophagy in cancer cells by targeting ROS

Autophagy is a cellular mechanism that is essential for preserving homeostasis and metabolic equilibrium by targeting damaged organelles and nonessential proteins for lysosomal degradation. Despite its importance, the participation of autophagy in the advancement of cancer and its potential efficacy as a treatment target remain subjects of controversy (Kocaturk et al., 2019; Miller and Thorburn, 2021). Gong et al. (2014) have demonstrated that a modest concentration of shikonin (2.5 μM) triggered autophagy in hepatocellular carcinoma cells, a process indicated by the increased expression of LC3-II, the formation of acidic autophagic vacuoles, and the aggregation of GFP-LC3 punctae. Further investigation into the underlying mechanism indicated that shikonin treatment led to a rise in intracellular ROS levels, and the induction of autophagy could be mitigated by the application of ROS scavengers. Moreover, shikonin activated the ERK signaling pathway while suppressing the RIP pathway. *In vivo* experiments demonstrated that shikoninsuppressed the growth of hepatocellular carcinoma tumors and induced autophagy without eliciting apoptosis (Gong et al., 2014). Shikonin triggers autophagy within XPC-3 human pancreatic cancer cells by upregulating LC3B-II expression and downregulating p62, p-PI3K, and AKT levels (Shi and Cao, 2014). In A375 human melanoma cells, shikonin triggers a defensive autophagic response via the ROS and p38 signaling pathways (Liu Y. et al., 2019). What’s more shikonin facilitates the dimerization of galectin-1, thereby activating the JNK, which ultimately results in cell autophagy and apoptosis in colorectal cancer cells (Zhang et al., 2020). Cai’s team found that Shikonin elevates the levels of autophagy-associated proteins, such as LC3 and P62, in renal cell carcinoma (RCC) cells by producing ROS. Conversely, treatment with NAC attenuated the autophagic activity (Tsai et al., 2021).

#### 4.2.3 Shikonin targets ROS to induce necroptosis in cancer cells

The well-established biological features of apoptosis have fueled the development of numerous drugs designed to trigger cell death in cancerous cells. Recent investigations have shown that some cancer cell lines, particularly those from pancreatic and melanoma tissues, have become resistant to apoptosis. These cells exhibit diminished apoptotic rates, reduced responsiveness to chemotherapy, and mutations in genes encoding pro-apoptotic proteins. Consequently, the modulation of necroptosis, an alternative programmed cell death pathway that occurs in a caspase-independent manner, is emerging as a potentially effective strategy to circumvent apoptosis resistance in cancer therapy. Ye et al. have shown that shikonin induces the activation of MLKL The activated MLKL initiates chromatin breakdown by enabling the nuclear import of AIF and inducing the production of γ-H2AX, thus facilitating necroptosis in glioma cells. Furthermore, MLKL enhances the activation of upstream signals RIP1 and RIP3 by interfering with mitochondrial function and intracellular ROS enhancement, which potentiates the effects of shikonin-induced signaling (Ding et al., 2019). Liu and colleagues have identified that shikonin orchestrates necroptosis and autophagic flux disruption through modulation of the RIP3/p62/Keap1 complex regulatory system. By inhibiting autophagy through the downregulation of RIP3, shikonin enhances necroptosis. The synergistic use of shikonin with late-stage autophagy inhibitors can significantly amplify necrotic apoptosis by perturbing RIP3 degradation in bladder cancer cells, both *in vitro* and *in vivo* (Liu et al., 2023). Additionally, a clinical study exploring the application of shikonin in the treatment of melanoma cells has indicated that shikonin elicits its antineoplastic properties by inducing necroptosis in these cells. Western blot analysis further corroborated the occurrence of necroptosis, demonstrating the upregulation of proteins including Chop, RIP1, and Prip1 (Ahmad et al., 2023).

#### 4.2.4 Shikonin targets ROS to induce ferroptosis in cancer cells

Elevated intracellular iron levels combined with glutathione (GSH) depletion generate an oxidative stress burden that culminates in a lethal buildup of peroxidized polyunsaturated fatty acids (PUFAs), which is a hallmark feature of cell death mediated by iron. Shikonin modulates ROS to cause iron death in cancer cells, and Ni et al. (2023) found that shikonin conjunction with cisplatin overcame drug resistance in cancer cells, downregulated GPX4, and upregulated haemoglobin oxygenase 1 (HMOX1) inducing iron death in cells. Qian and colleagues have shown that shikonin may inhibit small cell lung cancer by triggering an ATF3-dependent ferroptosis mechanism. This involves an elevation in the levels of ROS, a reduction in the functionality of GPX4, and an enhancement in the concentrations of 4-HNE, a marker indicative of ferroptosis (Qian et al., 2023). Recently, the design model of Fe-shikonin supramolecular nanomedicine has harnessed metal-polyol coordination technology to enhance the bioavailability of shikonin while mitigating its toxicity towards normal tissues. Furthermore, this method synergistically combines the therapeutic and diagnostic capabilities of iron ions with shikonin. This groundbreaking strategy in nanodrug development has the potential to transform the landscape of disease treatment and diagnosis by capitalizing on the distinctive properties of both shikonin and metal ions (Ren et al., 2020; Cao et al., 2022). The research conducted by Feng and colleagues revealed that tumor cells exposed to moderate to high concentrations of glutathione (GSH) undergo the degradation of the developed nanodrugs into Fe^2+^ and Shikonin. The metabolic change prompts ferroptosis and necroptosis in cancer cells, which emphasizes the capability of nanodrugs to take advantage of the specific internal environment of tumor cells. By doing so, they can activate certain cell death mechanisms, which may boost the effectiveness of anti-cancer therapies (Feng et al., 2022).

#### 4.2.5 Shikonin inhibits the activity of topoisomerases

DNA topoisomerases, commonly referred to as Topoisomerases or Topo, are enzymes resident in the cell nucleus that regulate the topological configuration of DNA by facilitating the cleavage and ligation of DNA strands (Pommier et al., 2022). DNA TOPO are crucial for converting supercoiled DNA to a relaxed state, and tumor cells, which continuously replicate DNA, are particularly vulnerable to these enzymes. This vulnerability presents an opportunity for developing therapeutic approaches that target tumor cell replication (Yakkala et al., 2023). Currently, DNA TOPO are also widely used as anticancer targets, such as irinotecan and trastuzumab (Bailly, 2019; Keam, 2020). Acetylshikonin was identified as a TOPO I inhibitor in 1995 (Ahn et al., 1995); In 1998, Plyta and colleagues discovered that alkannin and naphthoquinones both act as inhibitors of TOPO I (Plyta et al., 1998). A review article from 2023 also provided a detailed account of the use of shikonin and its derivatives as TOPO inhibitors in the development of anticancer formulations, owing to their dual TOPO inhibitory activity and minimal side effects (Olatunde et al., 2023). In a study spearheaded by Zhou, *in vitro* and *in vivo* models were constructed. *In vitro* experiments revealed that shikonin showed varying degrees of efficacy against different glioma cell lines, with IC50 values of 6 μmol/L, 4.35 μmol/L, 9.97 μmol/L, and 10.0 μmol/L for C6, SHG-44, U87, and U251 cells, respectively, and the minimum threshold of activity was determined to be 1 μmol/L. *In vivo* experiments, nude mice were administered shikonin at a dose of 2 mg per kg of body weight. Shikonin at a dose of 2 mg per kg body weight, the tumour volume and weight of subcutaneously injected gliomas in mice treated with shikonin were significantly reduced compared with those in the control group. Western blotting analysis showed that shikonin significantly increased the expression levels of RIP1, RIP3, γ-H2AX, and phosphorylated ATM, which indicated that RIP1/RIP3 was induced activation and DNA double-strand breaks occur in the organism. Furthermore, inhibition of RIP1 or RIP3 expression significantly attenuated shikonin-induced DNA damage, suggesting that RIP1 and RIP3 play a key role in mediating this process by regulating ROS levels. Administration of the ROS scavenger NAC was effective in alleviating shikonin-induced DNA breakage, providing convincing evidence that ROS is a key inducer of DNA breakage under these experimental conditions (Zhou Z. et al., 2017).

#### 4.2.6 Shikonin targets ROS to induce cells to undergo cell cycle arrest

**FIGURE 8:**
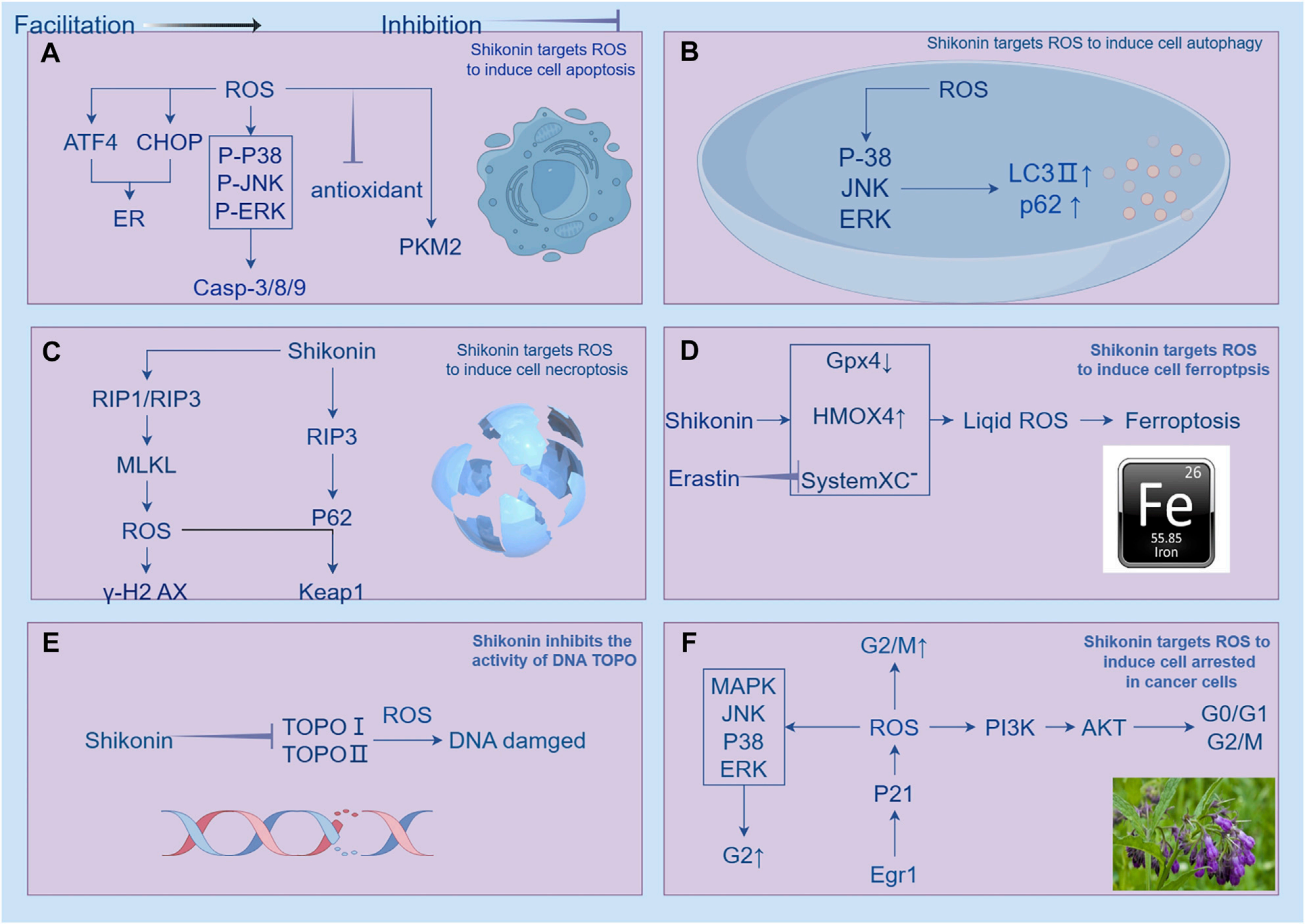
Shikonin has been shown to regulate ROS (reactive oxygen species) to induce cell death in cancer cells, which encompasses apoptosis, autophagy, necroptosis, ferroptosis, inhibition of topoisomerase activity, and cell cycle arrest. **(A)** illustrates how shikonin regulates ROS-induced apoptosis in cancer cells through mitochondrial-related, ER (endoplasmic reticulum)-targeted, and energy metabolism pathways. Shikonin promotes tumor cell apoptosis by increasing ROS production. Ginsenosides activate caspase 9/3 pathways and mitochondria-mediated apoptosis pathways in a ROS-dependent manner, and also induce mitochondria-dependent apoptosis by upregulating the ROS-mediated PTEN/PI3K/Akt/mTOR signaling pathway. Increased ROS also stimulates the upregulation of ATF4 (activating transcription factor 4) and CHOP (C/EBP homologous protein), interfering with glycolysis pathways. Reduction of ROS induced by shikonin also mediates apoptosis, with the upregulation of ROS-induced c-Jun N-terminal kinase (JNK) activity mediating mitochondrial apoptosis pathways, while inhibiting antioxidant enzyme activity also suppresses mitogen-activated extracellular signal-regulated kinase (MEK) and mitogen-activated protein kinase (MAPK). **(B)** shows that shikonin triggers autophagy in various cancer cell lines by increasing intracellular ROS levels, activating ERK and JNK signaling pathways, regulating the expression of LC3 and p62, and promoting the expression of autophagy-related proteins. **(C)** indicates that shikonin can coordinate cell necroptosis and autophagy flux disruption by modulating the RIP3/p62/Keap1 complex, enhancing cell necroptosis. **(D)** illustrates how shikonin downregulates GPX4 and upregulates heme oxygenase 1 (HMOX1), and Erastin increases lipid reactive oxygen species by inhibiting System Xc- to induce ferroptosis. **(E)** demonstrates how shikonin and its derivatives act as inhibitors of topoisomerase I (TOPO I), inducing RIP1/RIP3 activation and increasing intracellular ROS to induce DNA double-strand breaks. **(F)** illustrates how shikonin arrests the cell cycle at different stages, inducing cell death. Shikonin causes cell cycle arrest at G0/G1 or G2/M by reducing the expression of PI3K, p-PI3K, Akt, p-Akt, and m-TOR. It also inhibits cell proliferation by activating the Egr1-mediated p21 signaling pathway, leading to G2/M cell cycle arrest.

The cell cycle is essential in the development of cancer, which comprises interphase and the mitotic phase (M phase), regulates cell growth. Interphase is comprised of three phases: G1 phase, S phase, and G2 phase (Jamasbi et al., 2022). Cell proliferation is characterized by distinct phases within the cell cycle, with growth and DNA synthesis preparation occurring during G1, DNA replication during S phase and protein synthesis during G2 phase. The control of the cell cycle is managed by three key components: cyclins, cyclin-dependent kinases (CDKs), and cyclin-dependent kinase inhibitors (CKIs) (Wang, 2022). Isobutylshikonin exerts its inhibitory effect on HT29 cells by concentration-dependently decrementing the expression levels of PI3K, p-PI3K, Akt, p-Akt, m-TOR proteins, and their corresponding mRNA, resulting in the cell cycle arrest at the G0/G1 or G2/M phases (Zhong et al., 2018). The research conducted by Kim et al. (2014) has provided valuable insights into the mechanism underlying the anti-proliferative effect of shikonin on human gastric cancer cells (AGS). Their findings suggest that shikonin inhibits the proliferation of AGS cells by activating the Egr1-mediated p21 signaling pathway, which leads to G2/M-phase cell cycle arrest. Specifically, shikonin treatment increases the expression of Egr1, enabling it to directly bind to the promoter region of the p21 gene and induce its transcription, thereby triggering cell cycle arrest. The study further demonstrates that the induction of p21 expression by shikonin is compromised when Egr1 expression is inhibited, indicating a critical role of Egr1 in this process. These results establish a clear link between the anti-proliferative effects of shikonin and the Egr1-p21 signaling pathway, providing a solid experimental foundation for the potential application of shikonin in gastric cancer treatment (Kim et al., 2014). Chen’s research team has demonstrated that paclitaxel can effectively inhibit the growth of PC-322 and DU145 cells in a dose-dependent manner. Flow cytometry experiments have shown that paclitaxel can dose-dependently cause PC-3 and DU145 cells to accumulate in the G2 phase, while the proportion of cells in the G1 phase significantly decreases. For example, the percentage of cells in the G2 phase increased from 19% to 31.72% in PC-3 cells and from 7.74% to 31.72% in DU145 cells. This indicates that paclitaxel inhibits the proliferation of PC-3 and DU145 cells by causing G2 phase cell cycle arrest and regulates cell migration. Its mechanism of action includes dose-dependently inducing the phosphorylation of ERK, MAPK, and JNK, while significantly increasing the phosphorylation levels of AKT and mTOR. Subsequent studies have found that paclitaxel stimulates the production of ROS, and this effect can be reversed after treatment with dithiothreitol (DTT), highlighting the key role of ROS in paclitaxel-mediated cellular responses (Chen et al., 2014). The study conducted by Ahn et al. (2013) thoroughly investigated the underlying mechanisms of apoptosis induced by shikonin. Their experimental data revealed that shikonin can trigger the ROS/Akt/ASK1 signaling pathway and reduce the expression of p21Cip1, ultimately leading to apoptosis. Importantly, p21Cip1 fulfills multiple roles in this process. Initially, during the early stage of shikonin treatment, the upregulation of p21Cip1 expression results in cell cycle arrest at the G1 phase, thereby inhibiting apoptosis induced by shikonin. Additionally, p21Cip1 can bind to pro-apoptotic molecules such as ASK1 and directly suppress the apoptotic process. As shikonin treatment duration increases, p21Cip1 expression is downregulated, and ASK1 is activated, eventually triggering apoptosis. Therefore, p21Cip1 functions as an inhibitor of the cell cycle, apoptosis, and a responder to oxidative stress in shikonin-induced apoptosis, making it a crucial regulatory molecule in the apoptotic process (Ahn et al., 2013) (Figure 8).

#### 4.2.7 The combination of shikonin with drugs can reverse drug resistance and enhance therapeutic efficacy

The efficacy of standalone therapies based on ROS generation for inhibiting tumor growth is often inadequate in clinical practice. However, when these therapies are integrated with conventional clinical drugs, they can not only broaden the utility of natural compounds in anticancer treatment but also mitigate their limitations through synergistic therapy approaches. The investigation conducted by Hong’s research group showed that a non-toxic level of acetylshikonin, when used in conjunction with TRAIL, markedly induced apoptosis in HepG2 cells, concomitantly elevating intracellular ROS levels. Additionally, this treatment regimen triggered DNA double-strand breaks in HepG2 cells. There was an upregulation of Bax and PUMA expression in the hepatocellular carcinoma (HCC) cells. Treatment with antioxidants such as NAC markedly diminished intracellular ROS levels and the cleavage of PARP-1, which inhibited PUMA expression in HepG53 cells, thereby impeding the apoptotic process (Hong et al., 2019). Chen and colleagues delved into the clinical utility of shikonin in conjunction with PD-1 therapy. Their findings suggested that shikonin’s inhibition of PKM2 activity induced a significant elevation of intracellular ROS levels. This increase in ROS subsequently boosted the expression of HSP70. Notably, the combined application of shikonin and PD-1 treatment substantially enhanced the recruitment and infiltration of CD8^+^ T cells into the tumor microenvironment, thereby altering the immune conditions within the tumor (Chen et al., 2024). Zhu et al., 2012 discovered that the synergistic use of shikonin and paclitaxel effectively reverses multidrug resistance in ovarian cancer cells by stimulating a considerable increase in ROS within the cancerous cells (Wang et al., 2019). Hu et al. (2020) showed that shikonin can augment the therapeutic impact of osimertinib on non-small cell lung cancer (NSCLC) cells with wild-type EGFR, accompanied by the production of ROS and the activation of ERS. Moreover, the suppression of ROS using NAC or GSH reversed the apoptotic and endoplasmic reticulum stress responses elicited by the combination of shikonin and osimertinib (Hu et al., 2020). Accordingly, although cancer cells exhibit a higher production of ROS compared to normal cells, excessive ROS generation can result in their programmed cell death. Consequently, methods that increase ROS generation have become an auspicious strategy in cancer therapy (Moloney and Cotter, 2017). Shikonin acts as a chemosensitizer for various anticancer drugs, such as cisplatin and arsenic trioxide, enhancing their efficacy by promoting the production of ROS (He et al., 2015; Song et al., 2016). Shikonin may overcome drug resistance and forestall the emergence of resistance (Bovan et al., 2024), Shikonin exhibits efficacy against both acquired resistance in diseases like gefitinib-resistant non-small cell lung cancer (NSCLC), afatinib-resistant NSCLC, and cisplatin-resistant ovarian carcinoma, as well as primary resistance in scenarios involving gefitinib- and erlotinib-resistant wild-type EGFR NSCLC (Li et al., 2017; Li B. et al., 2018; Shilnikova et al., 2018). The aforementioned studies have demonstrated that shikonin and its derivatives possess significant potential in enhancing the antitumor efficacy of conventional chemotherapeutic agents (such as paclitaxel), immunosuppressants (like PD-1), and immunotherapies (e.g., TRAIL). These studies have achieved this by promoting the production of ROS within tumor cells and upregulating the expression of apoptosis-related proteins, such as p53, PUMA, and Bax, ultimately leading to the induction of apoptosis in tumor cells. Despite variations in the mechanisms of action and research outcomes, these studies collectively corroborate the capacity of shikonin to augment the effectiveness of existing antitumor treatments through diverse pathways. However, these studies are limited in two ways: Depth and breadth of mechanism studies: although studies have proposed that shikonin may enhance the efficacy of chemotherapy or immunotherapy through a variety of mechanisms, these mechanisms have not been studied in sufficient depth and comprehensiveness. To better understand the mechanism of action of shikonin, more experimental studies are needed to provide sufficient data support and to elucidate in depth how shikonin modulates these pathways. Feasibility study for clinical application: Currently, the studies of shikonin in combination with chemotherapy or immunotherapy are mostly at the cellular level and in animal models, lacking the support of clinical data. In order to assess the actual efficacy and safety of comedicin combination therapy, clinical trials, including randomised controlled trials, are needed to provide more reliable data. Therefore, future studies should focus on in-depth investigation of the molecular mechanism of shikonin and clinical trials to validate its value in clinical treatment and to assess the potential risks and benefits of its use in combination with other drugs. These studies will provide a more solid scientific basis for the clinical application of shikonin in tumour therapy (Table 1).

**TABLE 1:** *In vivo* use of shikonin in tumour therapy.

Cancer type	Animal model	Dosage	Theraputic outcomes	Ref
Primary effusion lymphoma, PEL	GTO cells-bearing PEL xenografted Nude-RJ mouse mod	Two groups of mice were given intraperitoneal injections of 2.5 mg/kg SHK and PBS twice a week for 25 days	Spleen weight and ascites volume were significantly reduced in the SHK-treated mice, with statistically significant differences compared with the control group	Alam et al. (2021)
Colorectal cancer	HCT-116 and HCT-15 cells-bearing BALB/c mice	A treatment regimen of 3 injections per week for 2 weeks was administered at a dose of 3 mg/kg per injection	Shikonin significantly inhibited the growth of subcutaneous graft tumours in HCT-116 and HCT-15 cells, with inhibition rates of 52.3% and 67.8%, respectively	Qi et al. (2022)
Hepatocellular carcin	Huh7 cells-bearing male BALB/c SPF nude mice	Different doses of purslane (2.5 mg/kg and 5 mg/kg) or carrier were given by daily gavage for 3 weeks, respectively	The shikonin-treated group showed a significant reduction in tumor weight compared to the control group. The 5 mg/kg dose group had a mean tumor weight of 2.34 g, with an inhibition rate of 42%, while the 2.5 mg/kg dose group had a mean tumor weight of 1.89 g, with an inhibition rate of 25%	Gong et al. (2014)
Bladder cancer	EJ cells-bearing nude mice	Shikonin group (15 mg/kg/d and 30 mg/kg/d), CQ group (1 mg/kg/d), Shikonin + CQ group (15 mg/kg/d and 30 mg/kg/d). Among them, CQ was injected 1 h before Shikonin and administered every 2 days	Shikonin significantly inhibited the growth of EJ cell transplantation tumours, and the inhibitory effect was further enhanced by the combination of shikonin with CQ.Shikonin showed no significant toxicity in nude mice at the doses of 15 mg/kg/d and 30 mg/kg/d	Liu et al. (2023)
Ovarian cancer	A2780/DDP cells-bearing male BALB/c mice	3.0 mg/kg cisplatin and 0.8 mg/kg vincristine in combination every 3 days for 16 days	The combination of cisplatin or vincristine significantly inhibited the growth of subcutaneous graft tumours and reduced the size of tumour nodules compared with cisplatin or vincristine alone. Serum biochemical index tests also showed that the doses of the drugs used did not significantly affect the liver and kidney functions of the mice	Ni et al. (2023)
Glioma	C6 glioma cells-bearing nude mice	Control (0.9% NaCl) and shikonin (2 mg/kg body weight) treatments were given every 2 days for a total of four doses	Tumour volume and weight were significantly reduced in mice treated with shikonin. The expression levels of RIP1, RIP3, γ-H2AX and phosphorylated ATM were significantly upregulated in the tumour tissues of the shikonin-treated group	Zhou et al. (2017b)
Hepatocellular carcinoma	HepG2 cells-bearing nude mice	The treatment group was injected with vincristine acetate (1 μg/g bw) and TRAIL (100 ng/g bw) every 2 days for a total of 4 weeks; the control group was injected with the same volume of solvent	Combined treatment with acetylshikonin and TRAIL significantly reduced the number of tumour nodules in the livers of mice, with the number of tumour nodules in the treatment group being 2.7 times that of the control group. There was no significant difference in body weight changes between the two groups of mice, suggesting that the combined treatment of acetylshikonin and TRAIL had no significant cytotoxicity in mice	Hong et al. (2019)
Colorectal cancer	CT-26 cells-bearing female BALB/c SPF nude mice	Mice in the experimental group received peritoneal injections of 3 mg/kg of shikonin every 2 days for a total of 17 times. On days 5 and 9, mice also received 23 μg/litter of anti-PD-1 antibody intraperitoneally, respectively. Control mice received equal amounts of DMSO and IgG as controls	The tumour volume of mice in the anti-PD-1+Shikonin group was significantly smaller than that of the control group	Wang et al. (2019)

## 5 Nanomedicine perspectives in shikonin therapeutics

Shikonin is a hydrophobic plant polyphenol with multiple physiological activities (Sun et al., 2022), because of its low solubility and chemical stability in aqueous media, as well as its low oral bioavailability and widespread toxicity, the clinical utility of shikonin is constrained. Nanomedicine, however, provides several benefits such as extended circulation duration, increased medication bioavailability, controlled drug release, improved treatment outcomes, and less adverse effects (Farokhzad and Langer, 2009; Yan et al., 2023). The prodrug nanotherapy, Cu-SK@DTC-PPB, functions as a nanomagnifier for highly selective antitumor treatment by activating prodrugs. In an environment rich in ROS within the tumor microenvironment, DTC-PPB is activated, releasing a less cytotoxic diethyldithiocarbamate (DTC). This activation process facilitates the chelation of Cu2+ from the Cu-SK framework, leading to the synthesis of a highly cytotoxic Cu(DTC)2 and triggering the consecutive release of shikonin. The released shikonin can produce ROS, increasing the amount of intracellular ROS. This then further activates DTC-PPB, leading to the release of more DTC. This positive feedback loop strengthens the therapeutic impact, producing a synergistic effect that intensifies the antitumor activity (Huang et al., 2022). Guo and colleagues have created Zn-shikonin-PEG hybrid nanoparticles (Zn-Shik-PEG NPs) using an organic-inorganic hybridization strategy that involves metal-phenol coordination. These nanoparticles improve the water solubility and biocompatibility of shikonin. By regulating the AMPK/SIRT3 pathway to suppress inflammation and apoptosis induced by the NLRP1 inflammasome, these hybrid nanoparticles may function as a new treatment for diseases and malignancies linked to ROS (Guo et al., 2023). The HFM@SK@HA nanodelivery system, which incorporates shikonin and hyaluronic acid (HA)-modified hollow Fe-MOF (HFM) cores, serves as an effective inhibitor of glycolysis. This attribute not only augments the therapeutic efficacy of microwave (MW) hyperthermia but also catalyzes the generation of ROS from endogenous H_2_O_2_. Consequently, the application of MW irradiation on the HFM@SK@HA system *in vitro* has the potential to markedly amplify its antitumor activity (Chen et al., 2023). The interaction of Shikonin with silver nanoparticles enhances their stability, leading to a marked reduction in the viability and growth of A549 lung cancer cells, suggesting strong potential for more effective lung cancer treatment than single therapies (Fayez et al., 2020). Hence, the creation of a Shikonin-based delivery system is a promising strategy that could enhance the efficacy of anti-cancer treatments while minimizing associated risks and side effects.

## 6 Limitations

Taken together, there is an encouraging anticancer effect of shikonin in both cellular and animal models, However, preclinical studies on shikonin are scarce and the corresponding evidence is insufficient, possibly due to the following reasons: 1) Extraction and Purification: Natural extracts of shikonin have a relatively low concentration, necessitating a complex extraction and purification process to obtain a high-purity compound. Moreover, due to the thermodynamic instability of shikonin, rapid degradation occurs when the temperature exceeds 60°C during hot reflux and microwave-assisted extraction processes, which increases production costs and may lead to higher prices for the final drug (Huang et al., 2020). 2) Bioavailability issues: Shikonin is a hydrophobic natural molecule with suboptimal solubility, rapid intestinal absorption, and a distinct “first pass” effect, resulting in low oral bioavailability (Yan et al., 2023). For instance, when rats were intravenously administered shikonin, the half-life was found to be 15.15 ± 1.41 h, with a maximum plasma concentration (Cmax) of 0.94 ± 0.11 μg/mL (Shao et al., 2020). Notably, the detection of shikonin in the plasma was no longer possible at 10 h post-intravenous administration, when a dose of 1.5 mg/kg was used. Animal studies have revealed that shikonin exhibits a distinct “first pass” effect in rats, with the highest drug concentrations observed in the bile and liver (Li M. et al., 2018). Encouragingly, in view of the limitations of shikonin mentioned above, research on methods for their modification is emerging, with studies on chemical modifications that alter the structure of the shikonin backbone and the development of nanodelivery systems (Mitra and Dash, 2018; Long et al., 2023). Ultimately, such modifications may effectively enhance pharmacological activity and bioavailability, as well as the targeting of shikonin to better treat cancer. Another prerequisite for establishing the clinical use of shikonin is the completion of clinical trials, in a study led by Guo, shikonin treatment was administered to 19 advanced lung cancer patients refractory to surgery, chemotherapy, or radiotherapy. The trial yielded encouraging results, including a >25% reduction in tumor diameter, a 37% response rate, and a 47% 1-year survival rate. ImNRFtantly, patient quality of life was substantially improved (Guo et al., 1991). Future research on shikonin may focus on several key areas to enhance its development potential: 1. Investigation of Shikonin’s Inhibitory Effects on Diverse Cancers: While shikonin has demonstrated anti-tumor capabilities, its differential efficacy across various cancer types remains to be comprehensively elucidated. Prospective studies could employ *in vitro* and animal models to examine the inhibitory potential of shikonin on a spectrum of cancers, including lung, breast, liver, among others. 2. Evaluation of Shikonin’s Safety and Toxicity: A comprehensive assessment of shikonin’s long-term safety profile is essential to establish its candidacy for clinical use. Toxicity studies are necessary to ensure the agent’s safety for extended exposure and to provide a scientific foundation for its judicious application. 3. Assessment of Shikonin’s Survival and Prognostic Effects: Large-scale clinical trials are warranted to examine the survival rates and prognostic impacts of shikonin treatment on cancer patients. Such studies will inform on the potential clinical utility of shikonin and its place in the management of cancer. It is reasonable to believe that with continuous technological development in pharmaceuticals and biochemistry fields, additional methods will be developed to improve the bioavailability of shikonin at a low economic cost, which is of great significance to fully utilize natural resources for the benefit of the public.

## 7 Conclusion

The extensive literature highlights Shikonin as a promising agent in cancer therapy, showing considerable potential owing to its capacity to regulate the redox balance in cancer cells. The diverse functions of ROS in cancer are contingent on the context, differing based on the tumor’s genetic profile, the particular ROS species present, and the level and duration of ROS dosage. As with cancer chemotherapy, leveraging ROS against cancer cells demands equilibrium, underscoring the need for a more nuanced and strategic investigation into ROS dynamics. Shikonin and its associated derivatives effectively combat cancer by directly or indirectly regulating ROS via diverse cellular signaling pathways, thereby disrupting the redox balance of cancer cells. Their favorable toxicity profile and efficient absorption properties render them attractive candidates for cancer treatment research.
